# cmvIL-10 Stimulates the Invasive Potential of MDA-MB-231 Breast Cancer Cells

**DOI:** 10.1371/journal.pone.0088708

**Published:** 2014-02-10

**Authors:** Cendy A. Valle Oseguera, Juliet V. Spencer

**Affiliations:** Department of Biology, University of San Francisco, San Francisco, California, United States of America; Cincinnati Childrens Hospital Medical Center, United States of America

## Abstract

Cancer is the result of unregulated cell growth that leads to tumor formation, and in many cases, metastases. Although there are several risk factors associated with cancer, one area that remains poorly understood is the impact of infectious disease. Human cytomegalovirus (HCMV) is a member of the herpesvirus family that is highly prevalent in the population. HCMV usually causes clinical disease only in immune compromised individuals, but recent evidence suggests that HCMV may be strongly associated with some forms of cancer, particularly glioblastoma and breast cancer. We investigated the possibility that cmvIL-10, a viral cytokine with homology to human IL-10 that is secreted from infected cells, could act in a paracrine manner to alter the tumor microenvironment, induce cell signaling, and increase the invasive potential of cancer cells. We found that human MDA-MB-231 breast cancer cells express the IL-10 receptor and that exposure to cmvIL-10 results in activation of Stat3, a transcription factor strongly associated with enhanced metastatic potential and chemo-resistance. In addition, cmvIL-10 stimulated an increase in DNA synthesis and cell proliferation, protected MDA-MB-231 cells from etoposide-induced apoptosis, and also greatly enhanced chemotaxis toward epidermal growth factor (EGF). These results suggest a significant and wide-ranging role for cmvIL-10 in the progression of breast cancer and could have broad implications for the diagnosis and treatment of cancer in HCMV-positive patients.

## Introduction

Breast cancer is the second leading cause of cancer deaths in the United States [1]. Many cancer patients do not die from local complications of their primary tumor growth, but rather from the malignant spread of the tumor. Approximately 30% of patients diagnosed with a solid tumor already have a clinically detectable metastasis, and for the remaining 70%, metastases are continually being formed throughout the life of the tumor [2]. While there are recognized genetic, environmental, and behavioral risk factors associated with breast cancer, little is known about the connection between infectious agents and breast cancer development or progression.

Human cytomegalovirus (HCMV) is a widespread pathogen that infects more than 70% of the general population [3]. In most individuals, primary infection with HCMV is asymptomatic; however, serious symptoms can occur in patients with compromised immune systems. HCMV pneumonitis greatly impacts the morbidity and mortality of transplant recipients, and HIV patients are frequently diagnosed with severe HCMV retinitis [3]. HCMV can be transmitted from mother to child during pregnancy, and infection can result in serious congenital defects, including deafness, mental retardation, and other neurological deficiencies[4].

The possible relationship between HCMV and cancer has been investigated for some time. The development of more sensitive detection methods has recently shown a very strong link between HCMV infection and glioblastoma, prostate cancer, and breast cancer [5]–[9]. While HCMV is not generally regarded as an oncogenic virus, the term oncomodulation has been proposed to describe the increased malignancy associated with HCMV-infected tumor cells [10]. The molecular mechanisms for oncomodulation include cell cycle dysregulation by immediate early proteins IE1 and IE2 [11], which promote entry into S phase, as well as the activity of the UL97 protein which phosphorylates and inactivates tumor suppressor Rb [12]. Recent studies of human breast biopsy samples have revealed abundant expression of IE1 [9]. In addition, the HCMV UL36, UL37, and UL38 gene products all interfere with caspase function and convey resistance to apoptosis [13], [14]. HCMV-infected neuroblastoma cells have been observed to down-regulate adhesion molecules and exhibit increased motility [15]. In prostate cancer and glioma cells, HCMV infection resulted in increased migration and invasion that was dependent on phosphorylation of focal adhesion kinase (FAK) [6], [16].

The ability to evade recognition from the immune system is also essential for cancer cells, and HCMV is highly adept at manipulating the host immune system [17]. The cmvIL-10 protein is a homolog of human IL-10 encoded by the UL111A gene product of HCMV [18]. Despite having only 27% sequence identity to human IL-10, cmvIL-10 binds to the cellular IL-10 receptor (IL-10R) and displays many of the immune suppressive functions of human IL-10 [19], [20]. Interestingly, elevated levels of IL-10 are frequently detected in the serum of cancer patients and correlate with poor prognosis [21]–[24], suggesting that IL-10 may contribute to immune suppression and protect tumor cells from cytotoxic T lymphocytes by down-regulation of class I and class II MHC. *In vitro*, IL-10 has been found to promote resistance to apoptosis in human breast and lung cancer cell lines [25], [26]. Furthermore, constitutive activation of Stat3, the primary downstream activator associated with IL-10 signaling, correlates with poor prognosis in ovarian cancer and is considered a key factor in the development of metastasis and resistance to chemotherapeutic agents [27]. Given that cmvIL-10 retains many biological functions of human IL-10, including stimulation of B cell growth and activation of Stat3 in monocytes and dendritic cells [20], [28], [29], we investigated whether the viral cytokine might also induce changes in human breast cancer cells that could ultimately promote tumor metastasis.

## Results and Discussion

**Figure 1:**
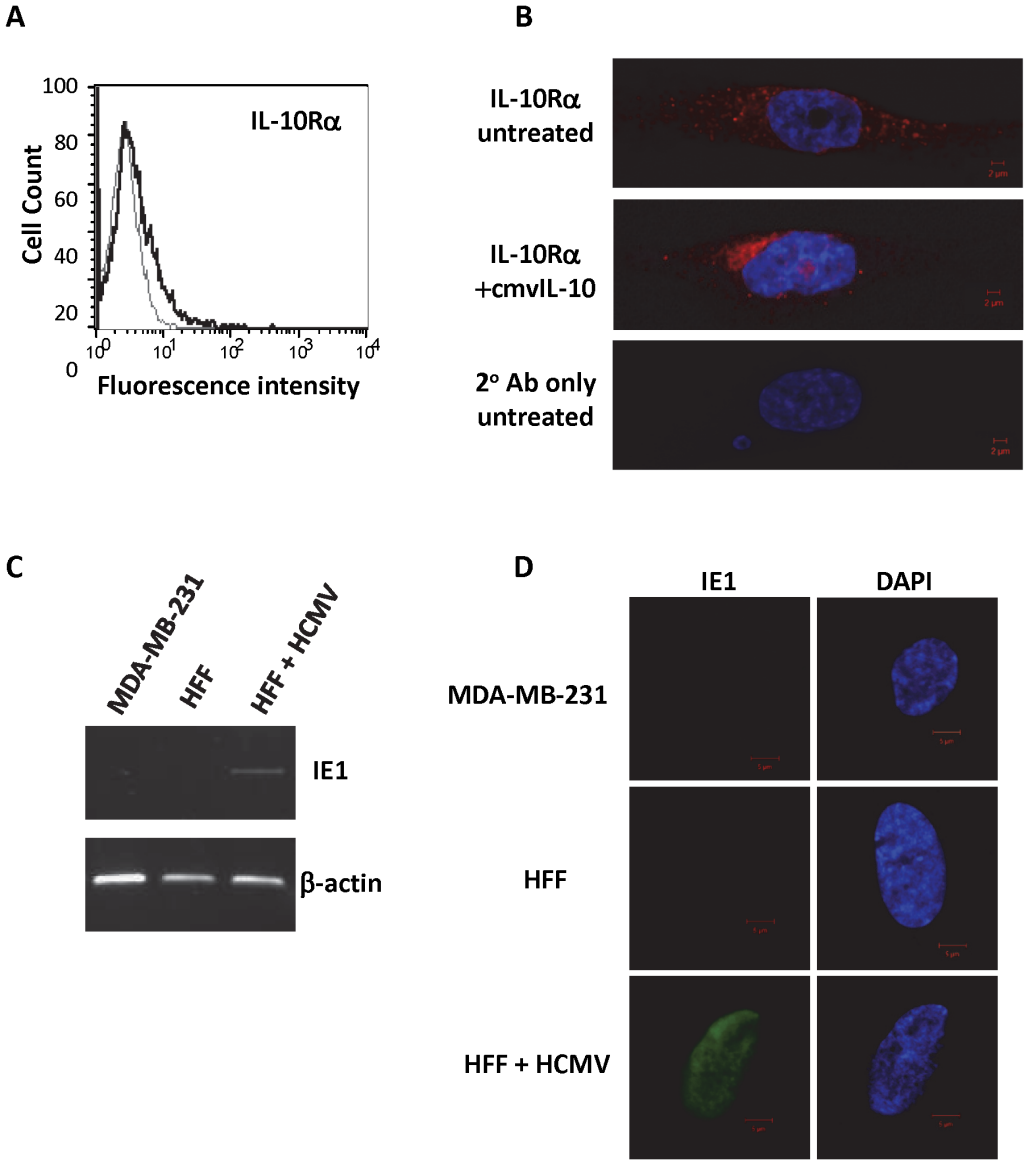
Human breast cancer cells express the IL-10 receptor. A) MDA-MB-231 cells were stained with anti-IL-10R-PE antibody (black line) or isotype control (gray line) and analyzed by flow cytometry. B) Cells were untreated or treated with 100 ng/ml cmvIL-10 for 15 min and stained for IL-10R followed by TRITC-conjugated secondary antibody, then visualized by fluorescence microscopy. Red corresponds to IL-10R, blue corresponds to DAPI staining of the nucleus. C) RNA was harvested from MDA-MB-231 cells and mock- or HCMV-infected HFF cells (MOI = 1, 72 hrs post-infection), reverse-transcribed and IE1 or β-actin gene specific primers were used for PCR. D) MDA-MB-231 and mock- or HCMV-infected HFFs were cultured on glass coverslips, fixed and stained for IE1 followed by FITC-conjugated secondary (green). These results are representative of three independent experiments.

In order to determine whether cmvIL-10 could have an impact on tumor cell physiology, we first examined whether breast cancer cells expressed the IL-10R. The MDA-MB-231 breast adenocarcinoma cell line was stained with antibody directed against the alpha chain of the human IL-10R complex and examined via flow cytometry. As shown in Figure 1A, there was low-level expression of the IL-10R complex detected on the surface of these cells. To study receptor distribution in greater detail, the cells were grown on glass cover slips, permeabilized, and visualized with immunofluorescence microscopy. The findings were consistent with the flow cytometry results in that the IL-10R complex was detected on the cell surface (Fig. 1B). However, additional receptor was also observed throughout the inside of the cell, suggesting that the IL-10R complex undergoes constitutive recycling in breast cancer cells and that surface levels are likely to be variable. After treatment with purified recombinant cmvIL-10, there was a distinct redistribution of IL-10R (Fig. 1B), indicating receptor internalization occurred rapidly after ligand engagement. Although HCMV infection has been noted in many primary breast tumor samples [9], [30], there was no evidence that the MDA-MB-231 breast cancer cell line was infected. Expression of the IE1 gene product could not be detected in these cells by RT-PCR (Fig. 1C) or immunofluorescence staining (Fig. 1D). Human foreskin fibroblasts that were infected with the AD169 strain of HCMV served as a positive control for IE1 expression, which was found to be localized predominantly to the nucleus, as expected (Fig 1D). These results demonstrated that uninfected tumor cells express the IL-10R complex and have the ability to respond to cmvIL-10 in the tumor microenvironment.

**Figure 2:**
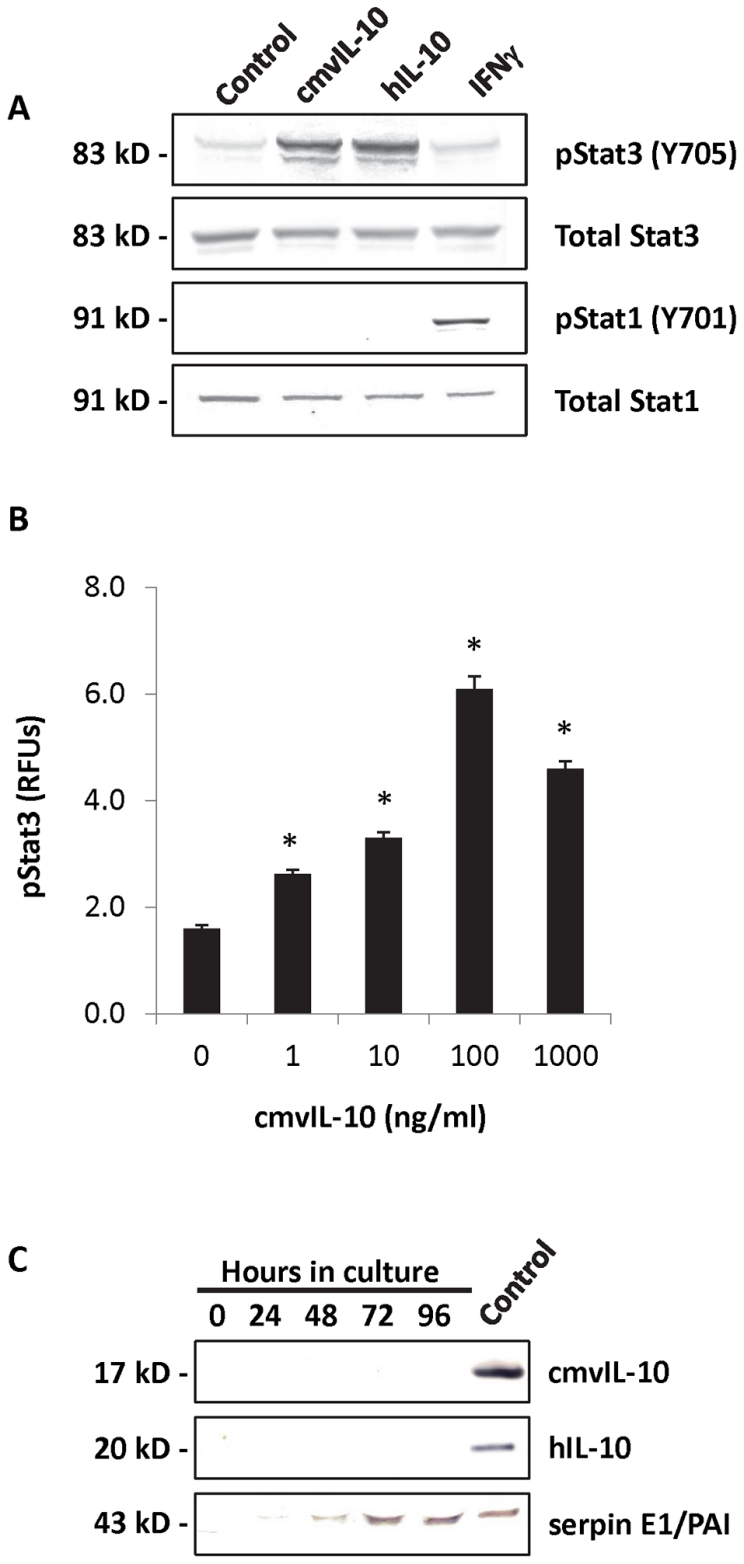
cmvIL-10 induces Stat3 phosphorylation in human breast cancer cells. A) MDA-MB-231 cells were treated with 100 ng/ml cmvIL-10, hIL-10, or IFNγ for 15 min, then lysed and Western blotted with the indicated antibodies. B) Cells were grown in 96-well dishes and treated with the indicated doses of cmvIL-10 for 15 min before lysis in the well followed by quantification of total vs. pStat3 levels. Results are represented as the normalized ratio of pStat3 to total Stat3 in relative fluorescence units (RFUs). *  =  p < 0.01, Student’s *t*-test. Error bars represent standard error for three replicates of each condition. C) MDA-MB-231 cells were cultured and supernatants collected at the indicated time points were subjected to SDS-PAGE followed by immunoblotting with the indicated antibodies. Control indicates purified recombinant protein (cmvIL-10, hIL-10, or serpin E1/PAI) was loaded as a positive control for each respective antibody. Results are representative of three independent experiments.

**Figure 3:**
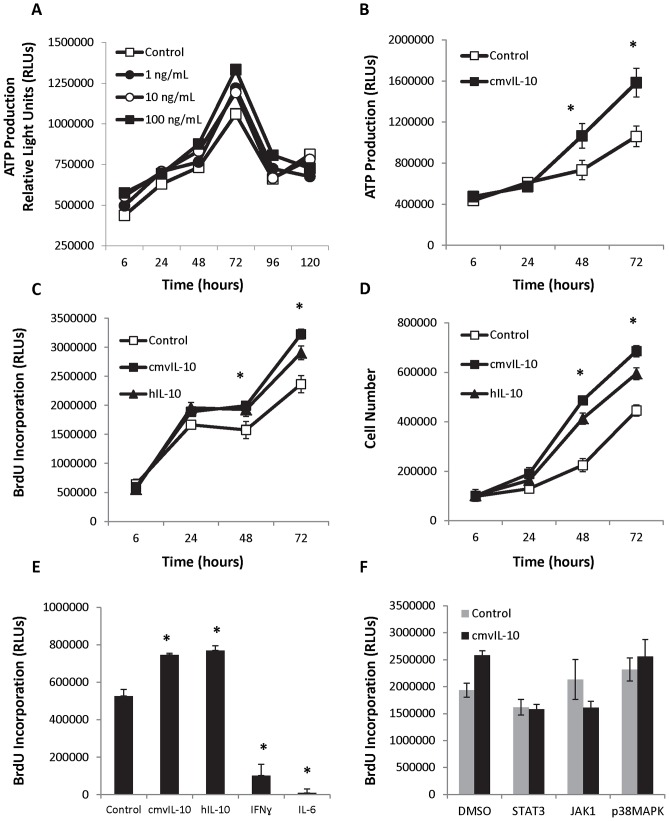
cmvIL-10 stimulates proliferation and increases DNA synthesis in human breast cancer cells. A) MDA-MB-231 cells were grown in 96-well dishes and treated with the indicated doses of cmvIL-10. Cell Titer Glo was added at the indicated time points to measure cell viability, represented as relative light units (RLUs) based on the resulting chemiluminescence. B) Cell growth in the presence or absence of 100 ng/ml cmvIL-10 via Cell Titer Glo. C) BrdU incorporation in the presence of 100 ng/ml cmvIL-10 or hIL-10. D) Standard cell counts of cultures from 6-well dishes containing 100 ng/ml cmvIL-10 or hIL-10 using a hemacytometer. E) BrdU incorporation was assessed at 72 hrs in cells cultured in the presence of 10 ng/ml of each indicated cytokine. F) Cells were treated with 10 µM of each indicated inhibitor or an equivalent volume of DMSO in the presence of absence of 10 ng/ml cmvIL-10 and BrdU incorporation measured after 72 hrs. Error bars represent standard error among three replicates for each condition. * indicates p<0.05, Student’s *t*-test. These results are representative of three independent experiments.

One of the earliest indicators of cmvIL-10 signaling is phosphorylation of Stat3 by the receptor-associated kinase JAK1. MDA-MB-231 cells were treated with either cmvIL-10, human IL-10 (hIL-10) or interferon-gamma (IFNγ), then cell lystates were examined by Western blot (Fig 2A). The expected 83 kD band corresponding to phosphorylated Stat3 (pStat3) was detected in cells treated with cmvIL-10 or hIL-10, but not in control cells exposed to PBS or IFNγ. Exposure to cmvIL-10 specifically activated Stat3, but did not globally activate other cellular effectors, such as Stat1, which was phosphorylated in response to IFNγ treatment only. To confirm Stat3 activation, cells were treated with varying doses of cmvIL-10 and then a cell-based ELISA was performed to detect pStat3. The amount of Stat3 phosphorylation increased in a dose-dependent manner with higher concentrations of cmvIL-10, as shown in Figure 2B. To confirm that Stat3 activation was solely due to treatment with cmvIL-10, MDA-MB-231 cells were examined for the production of endogenous hIL-10 by both ELISA (data not shown) and Western blot (Fig. 3C). No hIL-10 could be detected in the cell supernatants, confirming that Stat3 was not being activated by an autocrine signaling mechanism. Likewise, cmvIL-10 could not be detected in the supernatants of MDA-MB-231 cells (Fig. 1C), a result that was expected because we found that these cells were not infected with HCMV (Fig. 1C and D). Human serpin E1 (also known as PAI, plasminogen activator inhibitor-1), which is secreted from many cancer cells [31], served as a positive control and was detected in cell supernatants in increasing concentrations over time. Taken together, these results demonstrate that exogenous cmvIL-10 can trigger phosphorylation and activation of the transcription factor Stat3 in human breast cancer cells. Over-activation of Stat3 has been documented in glioblastoma, ovarian and breast cancers, which suggests that stimulation of this signaling pathway by cmvIL-10 could contribute to malignancy.

To elucidate downstream effects of cmvIL-10 signaling and Stat3 activation, cell proliferation was examined. MDA-MB-231 breast cancer cells were cultured in the presence of increasing doses of cmvIL-10, and cell growth was evaluated. Cell viability was measured by the addition of a luciferin substrate at the indicated time points, and the resulting luminescence is proportional to the amount of ATP present, reflecting the number of viable cells in the well. As shown in Figure 3A, cells exposed to cmvIL-10 exhibited greater growth than control cells. Overall cell growth increased for 72 hrs and then fell, possibly due to crowding in the wells. Subsequent assays utilized 100 ng/ml cmvIL-10 for 72 hrs, which resulted in significantly higher cell growth than control cultures (Fig 3B, *  =  p<0.05). In addition, BrdU incorporation was used to quantify the rate of DNA synthesis, which was found to be significantly higher in cells exposed to cmvIL-10 compared to the control cell lines (Fig. 3C). The level of proliferation induced by cmvIL-10 was comparable to that of hIL-10. Standard cell counts taken at each time point also revealed that cultures treated with either cmvIL-10 or hIL-10 had higher cell numbers than control cultures (Fig. 3D). The enhanced proliferative effect was specific to cmvIL-10 and hIL-10, as treatment with other cytokines did not increase cell proliferation. As shown in Figure 3E, treatment with IFNγ or IL-6 actually inhibited cell growth. Finally, treatment of cells with either a Stat3 or Jak1 inhibitor blocked the proliferative effects of cmvIL-10, confirming that these results are mediated in part by the Jak1/Stat3 signaling cascade. These results clearly demonstrate that cmvIL-10 specifically stimulates cell proliferation and increases the rate of DNA synthesis in human breast cancer cells.

**Figure 4:**
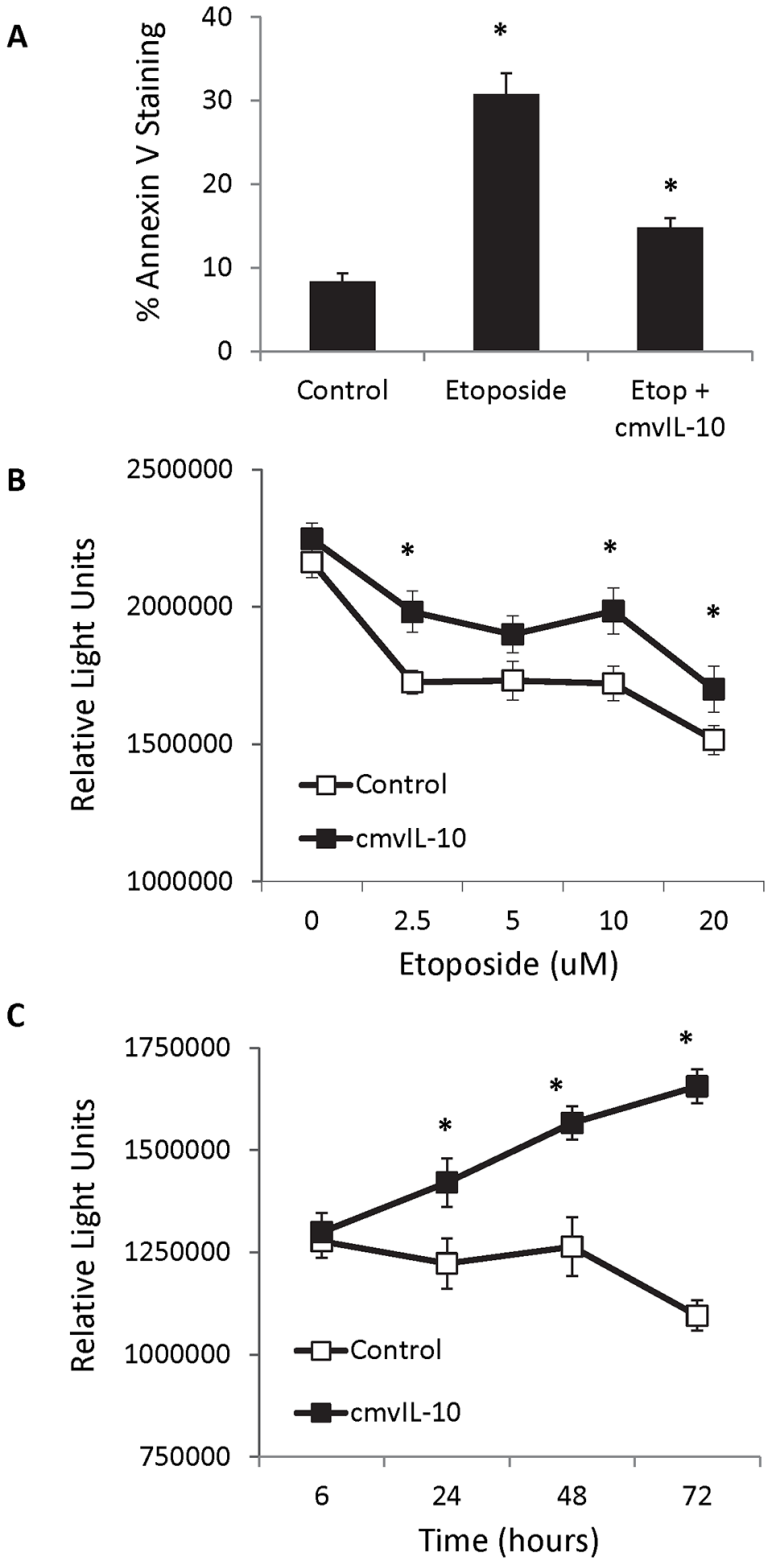
Human breast cancer cells are protected from apoptosis by cmvIL-10. A) MDA-MB-231 cells were treated with 100 µM etoposide in the presence or absence of 100 ng/ml cmvIL-10 for 48 hrs, then stained for Annexin V and analyzed by flow cytometry. B) MDA-MB-231 cells were grown in 96-well dishes and treated with the indicated doses of etoposide in the presence or absence of 100 ng/ml cmvIL-10 for 48 hours, then cell viability evaluated via the addition of Cell Titer Glo and detection of resulting chemiluminescence. C) Cells were cultivated in the presence of 10 µM etoposide with or without 100 ng/ml cmvIL-10. Cell viability was evaluated at the indicated time points via Cell Titer Glo. Error bars represent standard error of three replicates per condition. * indicates p<0.01, Student’s *t*-test. These results are representative of two independent experiments.

To investigate whether cmvIL-10 could protect cells from apoptosis, MDA-MB-231 breast cancer cells were treated with etoposide, an inhibitor of topoisomerase II that is widely used in the treatment of cancer based on its ability to induce cell death. After exposure to etoposide, 30.9% of cells stained positive for Annexin V via flow cytometry, as shown in Figure 4A. In contrast, when cultures were incubated with cmvIL-10 prior to etoposide treatment, only 14.8% of cells stained positive for Annexin V, indicating that cmvIL-10 was able to prevent induction of apoptosis in human breast cancer cells. Cultivation of cells with varying doses of etoposide revealed that cmvIL-10 increased overall cell viability (Fig. 4B), and cells exposed to cmvIL-10 were able to overcome etoposide-induced effects over time and proliferate robustly (Fig. 4C).

**Figure 5:**
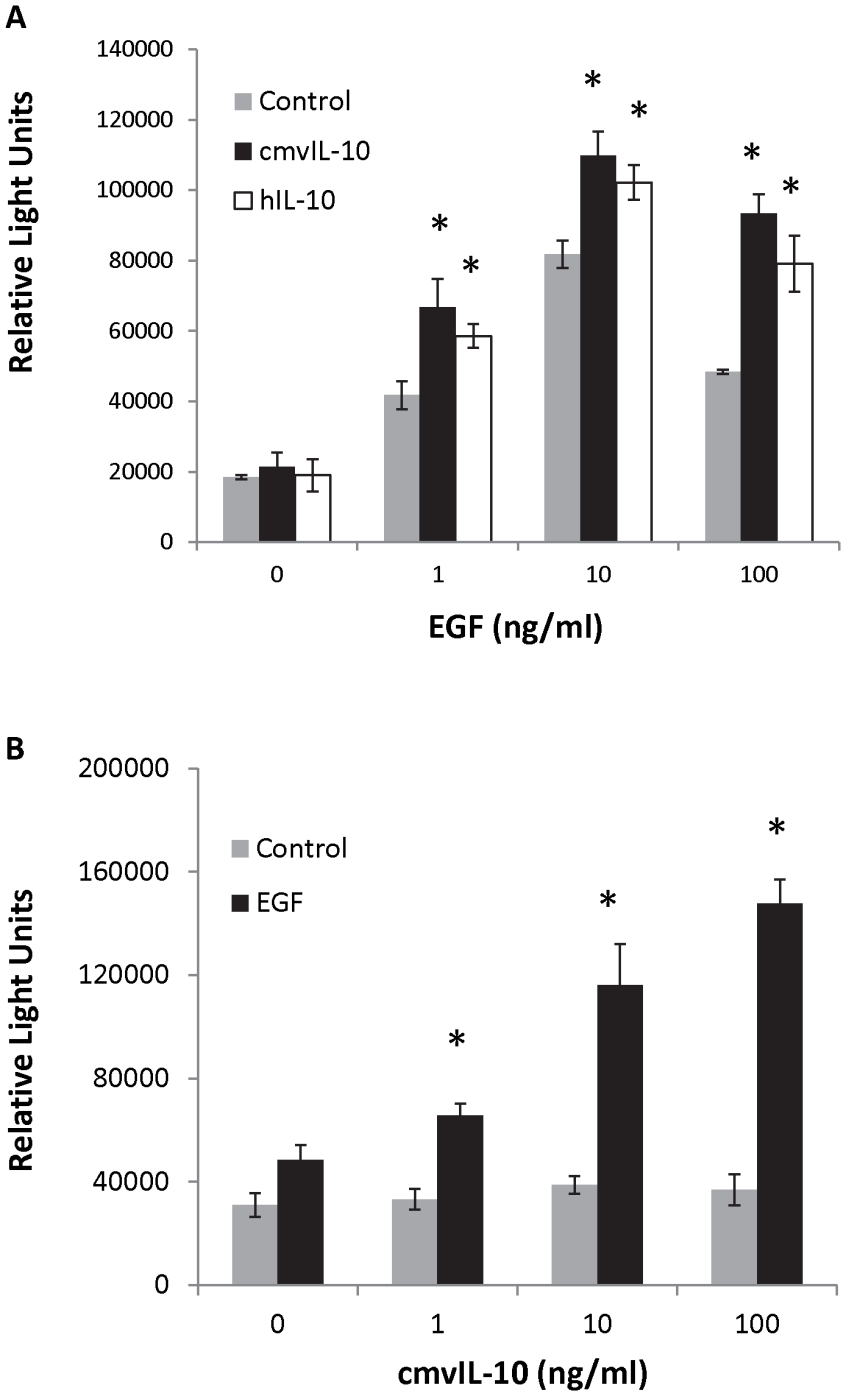
Human breast cancer cells exhibit enhanced chemotaxis when exposed to cmvIL-10. MDA-MB-231 cells were seeded at a density of 2×10^5^ cells in a total volume of 0.1 ml in the upper chamber of an 8 µm trans-well filter. A) Complete media containing the indicated concentrations of EGF in the presence or absence of 100 ng/ml cmvIL-10 or hIL-10 was placed in the lower chamber. After 5 hrs, cells in the lower chamber were harvested and quantified by the addition of Cell Titer Glo to measureme luminescence. B) Complete medium containing the indicated concentrations of cmvIL-10 in the presence or absence of 1 ng/ml EGF. Cells traversing the filter after 5 hrs were quantified as described. Error bars represent standard error. * indicates p < 0.05, Student’s *t*-test. Results are representative of three independent experiments.

Because the migration of cancer cells away from the primary tumor is one of the critical early factors in the formation of metastasis, we next examined cell motility. MDA-MB-231 breast cancer cells express the epidermal growth factor (EGF) receptor; therefore, EGF was utilized as a chemo-attractant in a modified Boyden chamber assay. The cells were placed in the top chamber separated from the EGF in the lower chamber by a porous (8 µm) filter, and after 5 hrs cells that had traversed the filter into the lower chamber were harvested and quantified. The cells migrated toward EGF and exhibited a standard bell-shaped curve for chemotaxis with a maximal response at 10 ng/ml EGF (Fig. 5A, gray bars). When both EGF and cmvIL-10 were present in the lower chamber, the migration response was significantly increased (Fig. 5A, black bars), and the effect of cmvIL-10 was comparable to that of hIL-10 (Fig. 5A, white bars). cmvIL-10 alone did not stimulate cell movement (Fig. 5B, gray bars); however, exposure to the viral cytokine significantly enhanced cell migration toward EGF (Fig 5B, black bars). Exposure to hIL-10 alone also failed to stimulate cell movement (data not shown). These results demonstrate that can cmvIL-10 work synergistically with other growth factors and mitogens present in the tumor microenvironment, such as EGF, to promote increased cell movement.

The hallmarks of cancer, characteristics such as sustained proliferative activity, resistance to apoptosis, and the ability to invade surrounding tissues which distinguish cancer cells from normal cells, were first described by Hanahan and Weinberg in 2000 [32]. The list was subsequently updated in 2011 to include additional characteristics, most notably, the ability to evade the immune system [33]. cmvIL-10 is well-documented to function as an immune suppressive cytokine that inhibits both immune cell activation and inflammatory cytokine production [19], [20]. Here, we show for the first time that cmvIL-10 can also enhance other properties associated with tumor cells. While this study made use of MDA-MB-231 breast cancer cells that already exhibit robust proliferation, treatment with cmvIL-10 was able to promote a significantly greater rate of growth. The rapid proliferation of cells at the primary tumor site increases the chances that any individual cell or group of cells might break away and initiate metastasis formation. Although evidence suggests that viral DNA and proteins can be found in tumors, no evidence of infection of the MDA-MB-231 cell line was found here and the role that virus infection plays in tumor development remains unclear. HCMV infects a wide range of cell types, including monocytes and dendritic cells which are mobile, circulating throughout the body and entering various tissues. It seems very likely that infected cells could enter a tissue containing a developing tumor, secrete cmvIL-10, and ultimately stimulate progression toward malignancy.

In addition to increased growth, we detected rapid phosphorylation and activation of Stat3 in breast cancer cells. This is significant because Stat3 is a transcription factor whose activation blocks normal programmed cell death pathways [34]. Elevated levels of pStat3 have been detected in gastric cancer [35] and are also associated with enhanced metastatic potential and chemo-resistance in ovarian cancer [27], [36]. In addition, constitutive activation of pStat3 in breast cancer cells has been observed to lead to increased expression of the anti-apoptotic protein survivin [37], which is consistent with our observations that exposure to cmvIL-10 protected cells from etoposide induced apoptosis. Therefore, when HCMV-infected cells secrete cmvIL-10 into their microenvironment, the viral cytokine could potentially impact neighboring cells by activating the IL-10R pathway, causing overstimulation of Stat3, enabling tumor cells to grow uncontrollably and resist the induction of apoptosis.

The ability of cancer cells to invade other tissues and spread to distant organs is an often fatal characteristic of malignant tumors. One of the key steps in the process of invasion is cell motility. Our results demonstrate that while cmvIL-10 itself does not trigger cell movement, the viral cytokine can enhance movement toward other growth factors such as EGF. Subsequent studies will be necessary to investigate whether cmvIL-10 can truly promote invasion through the extracellular matrix and into surrounding tissues, which might be expected to involve not only motility, but production of proteolytic enzymes.

HCMV is endemic in the human population and may be associated with several malignancies. Although various HCMV gene products have been shown to alter the cell cycle or inhibit apoptosis of virus-infected tumor cells, the possibility that factors secreted from virus-infected cells could affect tumor progression has not yet previously been examined. We demonstrate for the first time a novel effect of a secreted viral cytokine, cmvIL-10, in enhancing the invasive potential of human breast cancer cells. Although only a single cell line was tested here, MDA-MB-231 cells are widely used for breast cancer research because they are an excellent model for tumor cells, and these results are likely to be relevant to other cell lines as well as primary tumor cells. These findings suggest that the presence of cmvIL-10 in the tumor microenvironment could promote tumor progression and stimulate formation of metastases. These findings could ultimately lead to new therapeutic strategies for HCMV-positive breast cancer patients that include administration of antiviral drugs or anti-cmvIL-10 neutralizing antibodies in concert with traditional chemotherapy.

## Materials and Methods

### Cells & Reagents

MDA-MB-231 human breast cancer cells (American Type Culture Collection, Manassas, VA) were cultured in L-15 Leibovitz's Medium (Corning, Manassas, VA) supplemented with 10% fetal bovine serum (Atlanta Biologicals, Flowery Branch, GA) and maintained at 37°C with atmospheric CO_2_ according to the suppliers instructions. Purified recombinant cmvIL-10, hIL-10, IFNγ, IL-6 and anti-cmvIL-10, anti-hIL-10, anti-IL-10R, and anti-serpin E1/PAI antibodies were purchased from R&D Systems (Minneapolis, MN). Total Stat3, pStat3 (Y705), total Stat1, and pStat1 (Y701) antibodies were from Cell Signaling Technology (Danvers, MA). The Stat3 inhibitor was from Santa Cruz Biotechnology (Dallas, TX), the Jak1 and p38 MAPK inhibitors were from Calbiochem/EMD Millipore (Billerica, MA). Etoposide was from Cayman Chemicals (Ann Arbor, MI) and purified recombinant human EGF was from Peprotech (Rocky Hill, NJ). The HCMV strain AD169 (ATCC) was propagated in human foreskin fibroblasts (HFF, also from ATCC), maintained in Dulbecco’s modification of Eagle’s medium (Corning) containing 15% fetal bovine serum.

### Flow Cytometry

Monolayer cultures of MDA-MB-231 cells were harvested via gentle scraping according to manufacturer’s instructions (R&D Systems), pelleted with centrifugation at 1000x g, and then resuspended in FACS buffer (PBS + 1% BSA + 0.1% NaN_3_) at a density of 4.0×10^6^ cells/ml. For each experimental condition, 100 ul of cell were placed into 96-well plates and stained on ice protected from light with phycoerythrin (PE)-conjugated anti-hIL-10Rα or goat IgG PE isotype control antibody. After 1 hr, the cells were washed three times, resuspended in FACS buffer, and then fixed with 2% paraformaldehyde solution. Cell suspensions were analyzed using a FACSCalibur and CellQuestPro software (BD Biosciences, San Jose, CA).

### Reverse Transcriptase-Polymerase Chain Reaction

RNA was isolated from MDA-MB-231, HFF, and HCMV AD169-infected HFF cells using the RNeasy Mini Kit (Qiagen, Valencia, CA) according to manufacturer's instructions, followed by cDNA synthesis using the iScript cDNA Synthesis Kit (Bio-Rad, Hercules, CA). Each PCR reaction contained cDNA template, primers, dNTP mix, Ex-Taq buffer, and Ex-Taq polymerase (Clontech, Mountain View, CA). The gene specific primers for IE1 were 5′-GTGAGTCCGAGGAGATGAAATG-3′ (forward) and 5′-CTCGTAGATAGGCAGCATGAAC -3′ (reverse) and for β-actin 5′-AAGAGAGGCATCCTCACC-3′ (forward) and 5′-TACATGGCTGGGGTGTTG-3′ (reverse). The reaction underwent the following protocol on a T100 Thermal Cycler (Bio-Rad): 94°C for 5 min followed by 35 cycles of 94°C for 30 sec, 61°C for 30 sec, 68°C for 30 sec, followed by 1 cycle of 68°C for 5 min, and a final hold at 4°C. The PCR products were visualized on a 3% agarose gel.

### Immunofluorescence Microscopy

MDA-MB-231 cells were seeded into 6-well dishes containing FBS-coated glass coverslips at a density of 2×10^5^ cells per well and then incubated for 48 hrs at 37°C. Cell monolayers were washed with PBS, fixed with 4% paraformaldehyde, then permeabilized with 0.2% (w/v) Triton X-100 followed by treatment with ice cold 50% methanol-50% acetone for 30 min. Cells were then blocked with PBS + 10% FBS for 1 hr at 37°C and stained with anti-IL-10Rα antibody (Santa Cruz Biotechnology) at a 1∶100 dilution for 1 hr at 37°C. Following three PBS washes, the coverslips were incubated with TRITC-conjugated secondary antibody for 1 hr, washed again, and then mounted on a glass slide using Prolong Gold anti-fade reagent with DAPI (Life Technologies, Grand Island, NY). HFF cells were also grown on coverslips as described, mock- or virus-infected, and stained with anti-IE1 (EMD Millipore) followed by FITC-conjugated secondary antibody. Images were acquired using a Zeiss LSM700 laser scanning confocal microscope using Zen Black software (Carl Zeiss, Inc., Oberkochen, Germany).

### Western Blot and ELISA

For western blotting, cells were treated with 100 ng/ml cmvIL-10, hIL-10, IFNγ or PBS for 15 min, then harvested into cell lysis buffer (150 mM NaCl, 20 mM HEPES, 0.5% Triton-X-100, 1 mM NaOV_4_, 1 mM EDTA, 0.1% NaN_3_). Lysates were clarified, proteins were separated via SDS-PAGE, and then transferred to a nitrocellulose membrane. Then membrane was incubated in blocking solution (5% milk + 1X TBS-T) for 1 hr, then probed with primary antibody at a 1∶1000 dilution (total Stat3 or pStat3, total Stat1 or pStat1) in blocking solution overnight at 4°C. After washing, the membranes were incubated with a 1∶2000 dilution of appropriate AP-conjugated secondary antibody and bands were detected using Western Blue stabilized AP substrate (Promega, Madison, WI). For analysis of secreted proteins, supernatants were collected at various time points, analyzed by SDS-PAGE and then immunoblotted as above for cmvIL-10, hIL-10, or serpin E1/PAI. For the Stat3 ELISA, cells were seeded into 96-well dishes at a density of 1×10^4^ cell per well, treated with varying doses of cmvIL-10 in triplicate for 15 min, and then lysed in the plate and assayed for total or pStat3 using the Cell-based Stat3 ELISA kit according to manufacturer’s instructions (R&D Systems). The detection of hIL-10 in supernatants from MDA-MB-231 cell cultures was performed using the IL-10 ELISA DuoSet kit as directed (R&D Systems).

### Cell Proliferation and Apoptosis Assays

Cells were seeded into 96-well dishes at a density of 1×10^4^ cell per well in complete medium with varying doses of cmvIL-10, and then cell viability measured at the indicated time points using the Cell Titer Glo Assay kit according to manufacturer’s instructions (Promega). DNA synthesis was measured in cells prepared in the same way using the BrdU Cell Proliferation ELISA Kit (Roche, Basel, Switzerland). For experiments using inhibitors, cells were cultivated in 96-well dishes as above with a final concentration of 10 µM inhibitor and BrdU incorporation evaluated after 72 hrs. For cell counts, cells were seeded into 6-well plates at a density of 2×10^5^ cells per well, harvested via trypsinization, and counted using a hemacytometer at the indicated time points. The TACS Annexin V-FITC Detection Kit (Trevigen, Gaithersburg, MD) was used to stain cells harvested from 70% confluent T75 flasks that had been treated with 100 µM etoposide for 48 hrs in the presence or absence of 100 ng/ml cmvIL-10. Cells were then analyzed via flow cytometry to detect fluorescence. For viability assays, Cell Titer Glo reagent was utilized to quantify cells that had been seeded into 96-well dishes at a density of 1×10^4^ cell per well in complete medium with varying doses of etoposide in the presence or absence of 100 ng/ml cmvIL-10 as indicated.

### Migration Assays

Cells were harvested and resuspended at a density of 2×10^6^ cells per ml in complete medium. A total volume of 0.1 ml cell suspension (2×10^5^ cells) was placed in the upper chamber of a ThinCert filter with 8 µm pores in a 24-well plate (Greiner Bio-One North America, Monroe, CA). A total volume of 0.6 ml of media plus the indicated concentrations of human EGF and/or cmvIL-10 or hIL-10 was added to the lower chamber of each well, and plates were incubated for 5 hrs at 37°C. Medium from the lower chamber was collected, used to rinse the bottom of the filter twice, and then centrifuged at 1000 rpm for 10 min. The cell pellet was resuspended in 0.1 ml media and transferred to a white 96-well plate. Viable cell number was quantified using the Cell Titer Glo Assay kit according to the manufacturer’s protocol.

### Statistical Analysis

Statistical analysis was performed using the two-tailed Student’s *t*-test.
